# The Perception of Operational Sex Ratios by Voice

**DOI:** 10.1038/s41598-017-18182-4

**Published:** 2017-12-19

**Authors:** John G. Neuhoff

**Affiliations:** The College of Wooster, 1189 Beall Ave, Wooster, OH 44691 USA

## Abstract

Adult sex ratios in a local environment are linked to a wide variety of reproductive behaviors in humans and other animals. When sex ratios are biased, the more numerous sex faces increased competition for mates and is more likely to yield to the sociosexual preferences of the less numerous sex. Despite widespread evidence of the relationship between sex ratios and behavior, we know little about whether or how sex ratios are encoded and perceived. In two experiments men and women showed perceived sex ratios that correlated with actual sex ratios after 1500 ms exposures to groups of simultaneous voices. However, men perceived more female voices than women did, and women perceived more male voices than men did. Women showed better accuracy than men, but only when sex ratios departed markedly from 50%. Increasing the number of simultaneous voices reduced accuracy, but only at extreme sex ratios. Talker age also significantly affected perceived sex ratios, suggesting that perceived operational sex ratios are adaptively linked to the reproductive viability of the local population. The results suggest that listeners automatically encode vocal sex ratio information and that perceived sex ratios are influenced by characteristics of the local population and characteristics of the listener.

## Introduction

In humans and other animals, biased adult sex ratios are linked to variation in a wide variety of behaviors including mate selection, parental investment, resource allocation, promiscuity, and birth rates^1–8^. When sex ratios are male-biased (more men than women), behaviors by both sexes reflect this bias. For example, men tend to show greater intrasexual competition and invest more in offspring. Women tend to be more selective in choosing a mate^9,10^. Monogamous relationships are also more prevalent, women marry younger, and men incur greater debt because of increased competition for mates^4,11,12^. When sex ratios are female-biased, monogamy declines, and men are less committed to offspring and long-term relationships^12,13^. Men choose to marry later in order to take advantage of more abundant short-term mating opportunities^14^. Women increase the intensity of female-female competition and tend to be more willing to engage in casual sex^2,10^.

However, despite widespread evidence of the relationship between sex ratios and human behavior, almost no work has examined whether individuals consciously encode sex ratio information in the local environment. It is also unknown whether knowledge of local sex ratios accumulates over time, or can be immediately perceived after brief exposures. One study suggested that adult sex ratios can be accurately scaled from very brief visual displays of up to 12 concurrently presented faces^15^. However, no individual differences were reported, and sex ratio estimates did not differ between old and young faces. This suggests that visually, observers may extract adult sex ratios (the number of males to females) but not operational sex ratios (the number of reproductively viable males to reproductively viable females).

Like faces, voices are salient environmental stimuli that can convey the sex and age of the talker. Because age is correlated with fertility (particularly in women), voices have the potential to convey information about reproductive viability^16^. As such, voices could provide information about local operational sex ratios. Thus, the central question investigated here is whether listeners can accurately scale sex ratios from briefly presented samples of simultaneous speech that vary in the percentage of male and female talkers. In addition, we examined the effects of talker age, listener sex, and number of simultaneous talkers.

## Sexual Selection and Human Voices

As with many other sexually dimorphic traits, differences between male and female voices have likely evolved because of the evolutionary pressures of mate selection and intrasexual competition^17^. Men exhibit clear adaptive preferences for high pitched feminine voices^18,19^, and women show analogous preferences for low pitched masculine voices^20–22^. Some work has also shown that men with more masculine voices exhibit greater body size, strength, physical aggressiveness, and have higher levels of testosterone^23–25^. The role of voice pitch in intrasexual competition is supported by work that shows even a brief exposure to a female voice that is higher than average in pitch causes increased thoughts of same-sex aggression in women who are primed with a romantic scenario^26^. Men use and perceive vocal cues as signals of dominance and raise or lower their voice pitch depending on whether they believe they are addressing other men who are more or less dominant respectively than they are^27,28^. More generally, vocal characteristics have been found to be reliable indicators of mate quality and reproductive success^24,29–35^.

## Sex Differences in Voice Perception

One of the strongest cues to the sex of a talker is voice pitch. Male voices sound lower in pitch because the fundamental frequency of the male voice is nearly half of that of the female voice^35–38^. In fact, the effect size for the difference in fundamental frequency between male and female voices is among the largest of all sexually dimorphic human traits^17^. Female voices also exhibit greater variability and range of fundamental frequency^24,35^. Thus, vocal pitch and changes in vocal pitch are primary cues to the sex of a voice^39^. In addition to differences in fundamental frequency, male voices also have lower and more closely spaced formants than female voices^40,41^. Listeners can use these sex-differentiated cues to determine the sex of a voice accurately, and in some cases can discriminate male and female voices on the basis of formant spacing alone^42–48^.

Given the dramatic differentiation between male and female voices, it should not be surprising that men and women can show reliably different responses to voices and that sex differences in the perception of voices generally vary in ways that support survival and reproduction^49^. For example, vocal dominance cues are accurately perceived by both men and women. However, women are more sensitive than men to the dominance cues present in female voices, suggesting sensitivity to intrasexual competition^18,50^. In addition, women show processing advantages for emotional vocal stimuli, are better at identifying familiar voices, and show greater sensitivity to emotional prosody^51–53^. Women are also more sensitive than men to vocal intensity change that has social significance^54^. Although men and women do not differ in sensitivity to the high-frequency components of male voices, women do show greater sensitivity to the low-frequency characteristics of male voices than do men^55^. The neural response to vocal sounds is also differentiated by the sex of the listener^56–58^. For men, male and female voices have been shown to activate distinct brain regions. Female voices produce stronger activation of the right anterior superior temporal gyrus. Male voices preferentially activate the mesio-parietal precuneus area^59^.

## Aging, Fertility, and Voice

The aging process changes the acoustic characteristics of the voice. Older voices have a slower speech rate, poorer breath management (number of breaths and breath pause duration), increased instability (shimmer and jitter), increased glottal noise, and changes in speaking fundamental frequency^60–64^. Speaking fundamental frequency for women declines with age, whereas that of men either remains constant or rises slightly^65–67^. Cepstral peak prominence, an indicator of voice quality, has also been shown to change with age^68,69^.

Listeners can use voice characteristics to reliably judge a talker’s chronological age, although there is a slight tendency to overestimate the age of younger voices and underestimate the age of older voices^45,60,70–76^. A recent meta-analysis showed that the correlation between perceived age by voice and chronological age is 0.85^77^. Because chronological age is correlated with fertility and reproductive value, listeners presumably have access to the available information that would allow probabilistic fertility estimates by voice.

Several studies have linked fertility and reproductive value with vocal production. For example, over the menstrual cycle, women’s voice pitch increases during high versus low-fertility intervals^78,79^. These vocal changes correspond with a significant increase in voice attractiveness ratings as the risk of conception increases^80^. Moreover, listeners at peak fertility age (20–50 yrs.) are better at age estimation by voice than either adolescent (9–15 yrs.) or elderly listeners (over 60 yrs.)^74^. Female listeners additionally show increased galvanic skin response and increased heart rate when presented with naturally cycling, high fertility female voices^81^. Longitudinal reproductive value over the lifespan can also be reliably assessed by voice^82^, and women use voice parameters to assess men’s reproductive characteristics including age, weight, and testosterone levels^25^.

## Summary Statistics and Ensemble Coding

When presented with an array of similar objects, we generally do not process the specific details of each object in the array. Instead, we use *ensemble coding* to extract statistical averages of the features in the array^83,84^. For example, when presented with an array of dots of different sizes or lines of different orientations, observers are very good at perceiving the mean size and orientation of the sets, respectively, while retaining little information about the size or orientation of any individual token in the set^83,85^. Similarly, when presented with an array of faces showing different emotional expressions, observers are very good at extracting summary statistics and reporting the mean emotional expression^86^.

Although there is strong evidence to support the perception of fertility, age, and sex by voice, nearly all of this evidence comes from the presentation of single isolated voices. A group of simultaneous voices would represent an auditory array from which listeners might be able to extract summary statistics (e.g., average age or the proportion of male to female voices). In fact, the perception of summary statistics for sets of auditory stimuli including the mean frequency of a group of tones have recently been reported^87,88^.

## Perceiving Operational and Adult Sex Ratios from Voices

The term “adult sex ratio” refers to the ratio of adult males to females in a local population. This ratio can be contrasted with “operational sex ratio” which refers to the number of reproductively viable males to reproductively viable females in a local population^89,90^. Age is typically a naturally limiting factor in operational sex ratios. Women over the age of 50 generally excluded from operational sex ratios, and while men can remain reproductively viable until late in life, there is a general decline in fertility rates as men age^91^.

The ability to extract summary statistics from a stimulus array suggests that listeners may be able to perceive either adult or operational sex ratios from simultaneously presented voices. The detection of adult sex ratios would simply require determining the percentage of male and female voices in a crowd. The detection of operational sex ratios would further require incorporating information about the age of the individual talkers. Single voices are well categorized by sex and age, and these are precisely the variables required to perceive operational sex ratios.

## The Current Study

In two experiments listeners heard groups of simultaneous voices and were asked to judge the percentage of male and female voices in the group. We examined the effect of the actual sex ratio of the voices, the effect of listener sex, and the effect of the age of the voices. We also manipulated the number of simultaneous voices that were presented between experiments.

Two lines of evidence suggest that we might expect sex differences in the perception of vocal sex ratios. The first comes from work on individual voice processing. Several studies have shown that women exhibit an advantage over men in processing individual voices^51–53^. Thus, if these advantages manifest in processing multiple simultaneous voices, we might expect a female advantage in the perception of operational sex ratios. The second comes from a study of summary statistics. Although there is little work that examines sex differences in the perception of summary statistics, a recent study using morphed faces showed that women are more accurate at perceiving the average identity of a crowd of faces^92^. Women also show advantages over men in processing individual faces^93–95^. Faces and voices can convey similar information about identity and emotional state. Thus, there may be a female advantage in perceiving operational sex ratios by voice.

We might also expect differences in estimating sex ratios based on the age of the voices. Previous work has shown that young adult voices are more salient than old voices and can produce greater voice-age aftereffects^96^. This salience might increase accuracy in the perception of sex ratios. Alternatively, because of the greater reproductive value represented by young adult voices, it could distort actual sex ratios and produce perceptual errors that might nonetheless be adaptive^97–100^.

Finally, we might expect that increasing the number of simultaneous voices would reduce overall accuracy in estimating sex ratios. Increasing the number of talkers in a multi-talker stimulus increases masking for any given voice in the display^101^. Thus, each voice would become less distinct, and cues to age and sex such as speaking rate, formant structure and breath management might be more difficult to discern. However, appropriate scaling of sex ratios might still be obtained by reliance on fundamental frequency.

## Experiment 1

In Experiment 1, we employed a summary statistics method^15,87,88^ and presented listeners with brief audio clips of conversational speech from five simultaneous talkers. The stimuli resembled background chatter at a cocktail party. Half of the clips contained talkers who were over the age of 40; half contained talkers who were under the age of 30. The percentage of male voices in each clip ranged from 0–100%. After hearing each clip, participants used a sliding visual analog scale to indicate the proportion of males and females that they heard in the stimulus.

### Methods

#### Sample Size

An a priori power analysis was conducted using G*Power 3.1^102^. Sample size was subsequently set at *N* = 240 (120 participants per sex group). This yielded a power of 0.85 to detect a moderate effect size (*f* = 0.15) at an alpha level of 0.05 for the between-groups measure of a mixed design ANOVA with two between-groups (listener sex) and six repeated measures (sex ratio). Power for all within-groups and interaction effects exceeded that of the between-groups portion of the ANOVA. Four participants who reported hearing difficulties were replaced.

#### Participants

The sample consisted of 240 participants (120 female) with an average age of 38.3 yrs. (SD = 12.0). All reported normal hearing, were recruited via Amazon Mechanical Turk (MTurk), and were paid $0.75. All participants completed the study online. A wide variety of research has shown that samples from MTurk are more diverse and more representative of target populations and that their reliability is as good as or better than that obtained from traditional undergraduate samples^103,104^. MTurk samples have also been employed in previous online auditory perception studies^105,106^.

#### Stimuli

Voice stimuli were extracted from the Buckeye Corpus of Conversational Speech^107^. The corpus contains digitized conversational speech from 40 talkers stratified for age (under thirty and over forty) and sex. Four 1.5 s speech tokens were extracted from continuous conversational speech for each of the 40 talkers. Stimuli were selected such that each 1.5 s speech token started with the beginning of an utterance. Individual speech tokens were submitted to Praat^108^ for analysis of mean fundamental frequency and amplitude variation. Mean *(SD)* fundamental frequency for each group of speakers was as follows: Young female 197 Hz*(*5*2)*, Young male 124 Hz*(54)*, Old female 184 Hz*(47)*, old male 117 Hz*(41)*. Thus, the difference between both male and female voices, for both the old and young samples was approximately eight semitones (eight adjacent notes on the piano) with the old voices overall one semitone lower than the young voices. All speech tokens were equated for overall intensity. Mean amplitude variation (in standard deviations) for each group was as follows: Young female 8.1 dB, Young male 8.1 dB, Old female 8.7 dB, Old male 8.3 dB. Individual speech tokens were randomly selected into 12 combinations of five simultaneous voices for each of six sex ratios (0%, 20%, 40%, 60%, 80%, 100%) with the stipulation that no talker could appear more than once in a given combination of voices. The combinations were then digitally mixed and saved as single 320 kbps mp3 files. Each participant heard eight randomly selected combinations from the pool of 12 at each of the six ratios for both old and young talkers in random order blocked by talker age.

#### Design and Procedure

All procedures were approved by The College of Wooster’s Human Subjects Research Committee. All methods were performed in accordance with the United States Health and Human Services Policy for Protection of Human Research Subjects. After providing informed consent, participants were presented with a computerized text-to-speech voice that asked them to adjust their volume to a comfortable listening level. As verification that they could hear the auditory stimuli, they were verbally instructed to type a code word into a response box. They were then told that they would hear brief audio clips of voices talking at once and that their task was to estimate the proportion of male and female voices that they heard by using a response slider (a visual analog scale that was pictured on the instruction screen). The scale was anchored on the left with “100% Male” and on the right with “100% Female”. The center of the scale was marked “50% Male/50% Female”. Participants could move a cursor to any position along the scale to indicate their perceived sex ratios. Positions along the scale were internally recorded as 0–100, but scale markings were not visible to the participants. They were then given three practice trials with two concurrent voices at sex ratios of 0%, 50%, and 100%. Each participant then heard a total of 96 experimental trials.

### Results

#### Correlations

To determine whether listeners could accurately scale vocal sex ratios, perceived sex ratios were averaged across trials and voice ages for each participant. This resulted in one perceived sex ratio for each of the six actual sex ratios that were presented to each participant. Pearson correlations were calculated for each participant between perceived and actual sex ratios (N = 6 for each individual correlation). Correlations ranged from 0.94 to 0.99 and all 240 were significant at the 0.05 alpha level. To obtain the average correlation, each coefficient was subjected to Fisher’s r to z transformation and then the z-scores were averaged. The inverse z to r transformation on this mean yielded a mean correlation of *r*_(5)_ = 0.99 between perceived and actual sex ratios across all participants, providing evidence that listeners are extremely sensitive to sex ratio information from voices after brief exposures.

#### Perceived sex ratios

**Figure 1:**
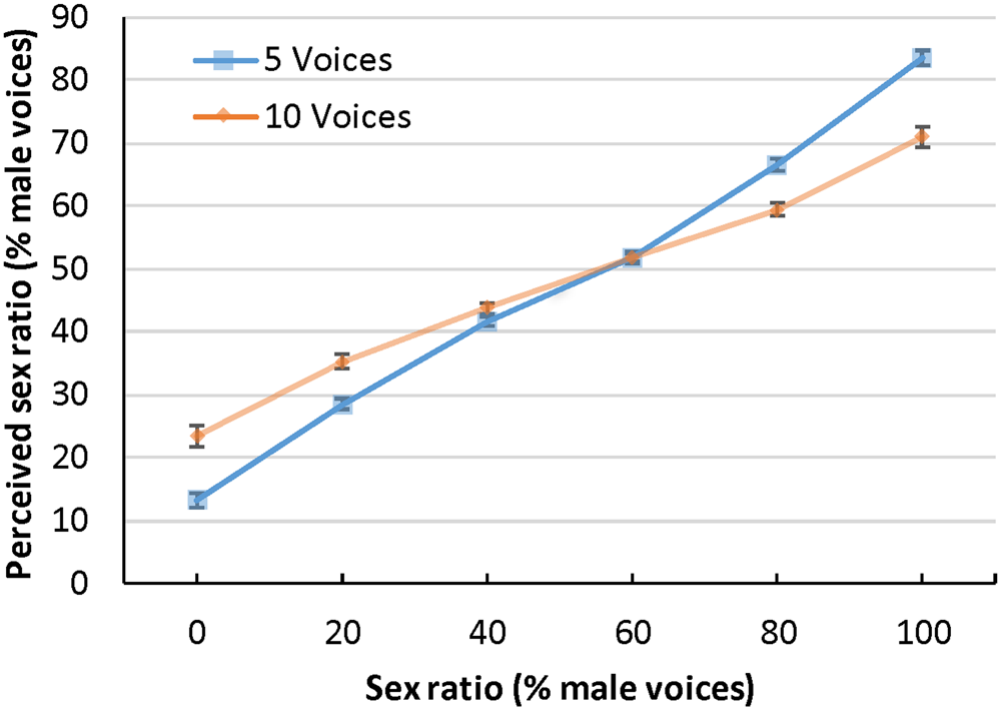
Perceived versus actual sex ratios for Exp.1 (five simultaneous voices) and Exp. 2 (ten simultaneous voices). Error bars represent 95% confidence intervals.

A 2 (talker age) × 2 (listener sex) × 2 (number of talkers) × 6 (sex ratio) mixed design ANOVA was conducted on the perceived sex ratio judgments and revealed a main effect for sex ratio *F*_(5, 1190)_ = 3289.2, p < 0.001,* η*_p_^2^ = 0.93 that showed that listeners could clearly discriminate among the different vocal sex ratios (see Fig. 1). The linear trend was significant *F*_(1,238)_ = 4577.69, p < 0.001 *η*_p_^2^ = 0.54. There was a main effect for listener sex indicating that men heard a smaller percentage of male voices (*M* = 46.81, *SE* = 0.38) than did women (*M* = 48.33, *SE* = 0.38), *F*_(1,476)_ = 8.12, p = 0.005 *η*_p_^2^ = 0.03. Follow-up one sample t-tests showed that both men, *t*(119) = 7.95, p < 0.001, *d* = 0.73, and women *t*(119) = 4.76, p < 0.001, *d* = 0.43, slightly underestimated the overall proportion male voices (50%).

There was also a significant main effect for talker age indicating that listeners heard a smaller percentage of male voices in the array of young talkers (*M* = 44.9, *SE* = 0.31) than in the array of old talkers (*M* = 50.22, *SE* = 0.31), *F*_(1,476)_ = 277.17, p < 0.001 *η*_p_^2^ = 0.54. Phrased differently, listeners heard a higher proportion of young female voices than old female voices. For the young voices, listeners significantly overestimated the actual percentage of females *t*(239) = 15.94, p < 0.001, *d* = 1.03. For the old voices there was no difference between actual and perceived sex ratios *t*(239) = 0.71, p = 0.48, *d* = 0.05. The significant main effect of a perceptual difference between young and old voices suggests that listeners are extracting operational sex ratios from the voices rather than simply adult sex ratios.

However, the interpretations of the significant main effects for ratio, age and listener sex are qualified by two significant interactions. There was a significant interaction between actual sex ratio and listener sex *F*_(5,1190)_ = 6.63, p < 0.001 *η*_p_^2^ = 0.03. Follow up Bonferroni-corrected t-tests showed that women heard more male voices than men did only when male voices were plentiful, at sex ratios of 80%, *t*(238) = 2.73, p = 0.007, *d* = 0.35, and 100% *t*(238) = 4.36, p < 0.001, *d* = 0.56. There was also a significant interaction between actual sex ratio and talker age, *F*_(5,1190)_ = 49.38, p < 0.001 *η*_p_^2^ = 0.17. Bonferroni-corrected t-tests showed that the percentage of male voices heard in the sample of old voices was higher than that heard in the young sample at each actual sex ratio. However, the effect sizes are greatest when the actual percentage of male voices is less than 50%. No other interactions were significant.

#### Error rates

**Figure 2:**
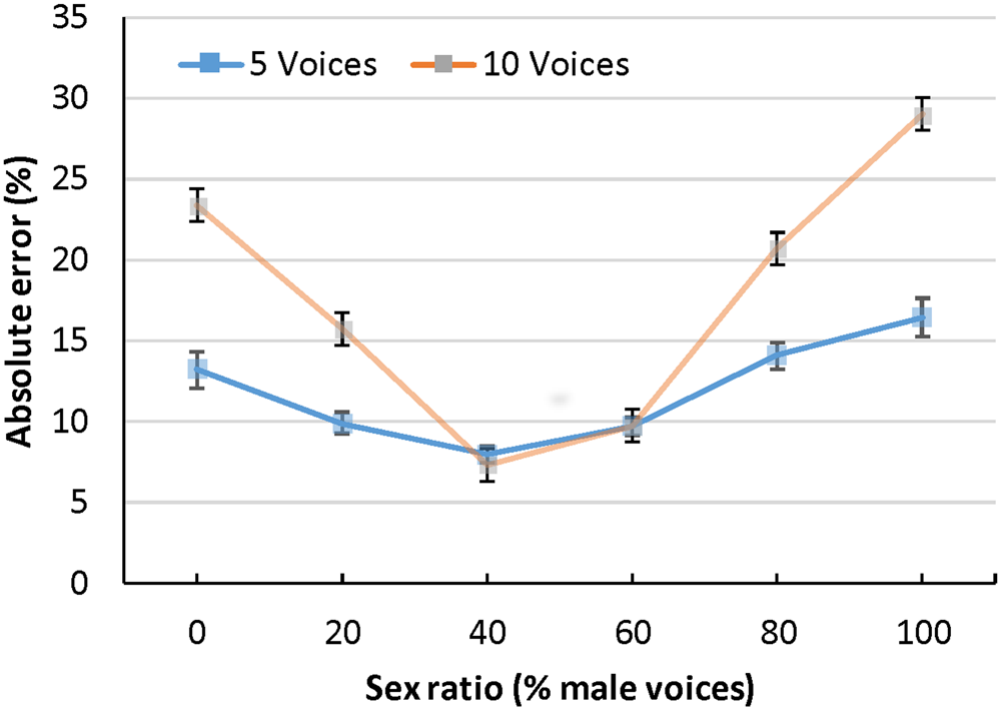
Absolute error rates for Exp.1 (five simultaneous voices) and Exp. 2 (ten simultaneous voices). Error bars represent 95% confidence intervals.

A second 2 (talker age) × 2 (listener sex) × 2 (number of talkers) × 6 (sex ratio) mixed design ANOVA was conducted on the absolute error rates for each participant. Absolute error was calculated by taking the absolute value of the difference between actual and perceived sex ratios in each condition. There was a significant main effect for sex ratio *F*_(5,1190)_ = 68.13, p < 0.001,* η*_p_^2^ = 0.22, that showed higher error rates as actual sex ratios departed from 50% (see Fig. 2). There was a significant main effect for talker age *F*_(1,238)_ = 14.52, p < 0.001 *η*_p_^2^ = 0.06 that showed higher error rates for older voices (*M* = 12.38, *SE* = 0.29) than for young voices (*M* = 11.38, *SE* = 0.29), and a significant main effect for sex *F*_(1,238)_ = 14.70, p < 0.001 *η*_p_^2^ = 0.06 that showed higher error rates for men (*M* = 12.84, *SE* = 0.36) than for women (*M* = 10.91, *SE* = 0.36). However, each of these main effects was again qualified by significant interactions.

**Figure 3:**
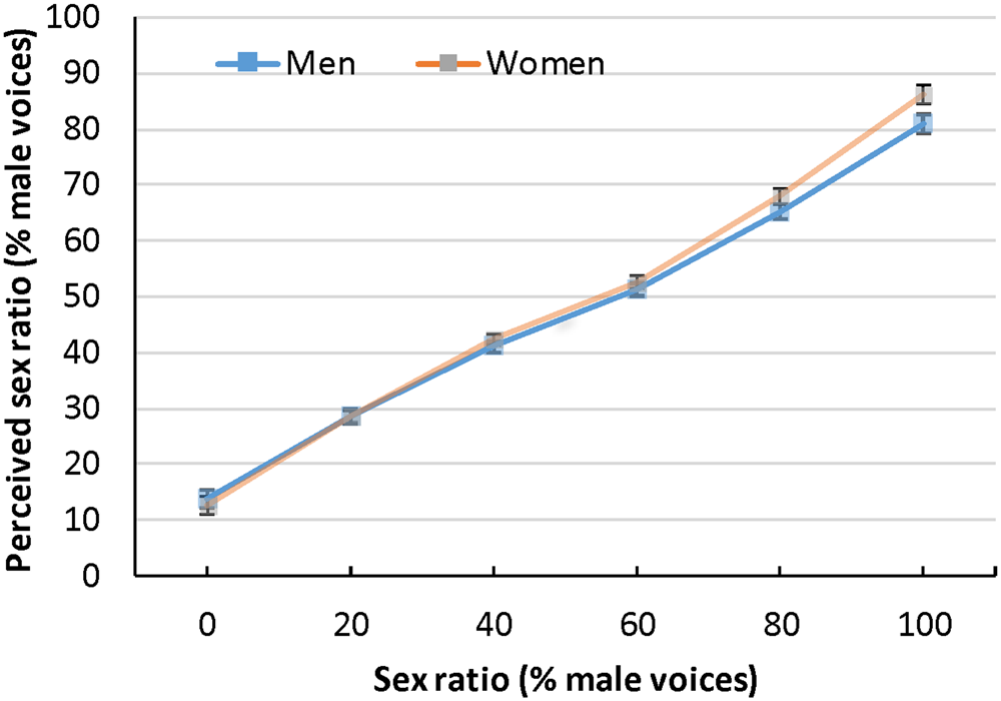
Perceived sex ratios for men and women in Exp. 1. Error bars represent 95% confidence intervals.

The interaction between actual sex ratio and listener sex was significant, *F*_(5,1190)_ = 5.90, p < 0.001 *η*_p_^2^ = 0.02. Follow up Bonferroni-corrected t-tests showed that women had lower error rates than men only when male voices comprised more than 50% of the array, specifically at sex ratios of 60%, *t*(238) = 2.72, p = 0.007, *d* = 0.35, 80%, *t*(238) = 3.31, p = 0.001, *d* = 0.43, and 100% *t*(238) = 4.63, p < 0.001, *d* = 0.56 (see Fig. 3). There was also a significant interaction between sex ratio and talker age, *F*_(5,1190)_ = 53.70, p < 0.001 *η*_p_^2^ = 0.18. Listeners had higher error rates for old voices when the actual percentage of male voices was below 50%, but higher error rates for young voices when the percentage of male voices was above 50%. No other interactions were significant.

### Discussion

The results of Experiment 1 show that listeners can accurately scale sex ratios of five simultaneous voices with exposures of only 1500 ms. Absolute performance was best for ratios close to 50% and was worst for extreme ratios of 100% and 0%. The compression of extreme sex ratios toward the 50% point may be due to greater experience with more sex-balanced ratios and is consistent with other vocal scaling tasks that show a regression to the mean at extreme values^77^. However, the experiment-wide absolute error rate of 11.9% is noteworthy given that there were only 5 voices in the array. The error rate of 11.9% suggests that listeners were precise in judging sex ratios to within 0.60 voices.

The sensitivity to talker age suggests listeners may be extracting operational rather than simply adult sex ratios. A key to this result appears to be the effect of young female voices. Across all ratios, listeners heard significantly more female voices in the array of young voices than in the array of old voices. This is indicated by a lower perceived sex ratio (% of males heard) for the array of young voices. The interaction between sex ratio and talker age shows that this effect is larger at low sex ratios (when there are more female voices in the array). Overall, listeners overestimated the percentage of young female voices but were accurate in perceiving the percentage of old female voices. This occurred despite a slightly greater separation in fundamental frequency between young male and female voices than between old male and female voices that in theory could have aided discrimination. These findings are consistent with previous work that suggests a particularly salient role for the characteristics of young female voices in the vocal perception of gender and gender stereotyping^109^.

The salient effect of female voices likely also explains why listeners of both sexes slightly underestimated the percentage of male voices in the array across age groups. However, the main effect for listener sex showed that averaged across all sex ratios, men heard more female voices than women did, and women heard more male voices than men did. This finding was primarily due to women perceiving more male voices than men did when male voices were plentiful (80% and 100% sex ratios). Although women still underestimated the number of male voices, they were more accurate than men. Examined from a different perspective, this means that men perceived more female voices than women did when female voices were scarce (20% and 0% female voices).

The data on absolute error show that women exhibited a slight but significant advantage over men in the accuracy of perceiving vocal sex ratios. This finding is consistent with previous work that shows a female advantage in voice processing and ensemble coding^18,50,52,53,55,92^. The significant interaction with sex ratio showed that the advantage occurred primarily at high sex ratios when female voices were in the minority and men overestimated the percentage of female voices more than women did. There was also a slightly higher error rate for old voices. The percentage of male voices was underestimated in the young sample more than in old sample. This finding again supports the salient effects of young female voices. However, these results should be interpreted in light of the significant interaction with actual sex ratio. At sex ratios above 50% (when men are plentiful) there was greater error for young voices. At sex ratios below 50%, there was greater error for old voices.

## Experiment 2

Experiment 2 provided a replication of Experiment 1 and examined the effects of increasing the number of simultaneous voices from five to ten. Increasing the number of simultaneous voices could obscure some of the acoustic cues that listeners might use to determine sex ratios. This would likely result in an increase in absolute error rates. However, if listeners are still able to scale sex ratios accurately, then the main effects and interactions found in Experiment 1 should replicate.

### Method

#### Participants

The sample consisted of 240 participants (120 female). Average age was 38.0 yrs (SD = 12.5). All reported normal hearing. They were recruited via Amazon Mechanical Turk (MTurk) and were paid $0.75. All participants completed the study online.

#### Stimuli

Voice stimuli were created in the same manner as in Experiment 1 with the exception that there were ten voices in each stimulus.

#### Design and Procedure

The design and procedure were the same as those used in Experiment 1.

### Results

#### Correlations

Pearson correlations were calculated for each participant between perceived and actual sex ratios as in Experiment 1. Correlations ranged from −0.74 to 0.99, and 215 of 240 were significant at the 0.05 alpha level. Five participants appeared to reverse the male-female scale (thus the negative correlations), though these correlations were not significant. Twenty participants had positive correlations that did not meet significance. A Fisher’s exact test showed that the proportion of participants with significant positive correlations in Experiment 1 (240/240) was significantly greater than the proportion in Experiment 2 (215/240), p < 0.001. Each individual correlation coefficient was subjected to Fisher’s r to z transformation, and then the z-scores were averaged to obtain the average correlation across participants. The inverse z to r transformation on this mean yielded a mean correlation of *r*_(5)_ = 0.97 between perceived and actual sex ratios across all participants. Although this mean correlation is extremely high, it is still significantly smaller than that obtained with five voices in Experiment 1 (*r* = 0.99), t_(238)_ = 5.53, p < 0.001, *d* = 0.50.

#### Perceived sex ratios

A 2 (talker age) × 2 (listener sex) × 2 (number of talkers) × 6 (sex ratio) mixed design ANOVA was conducted on the perceived sex ratio judgments and revealed a main effect for sex ratio *F*_(5,1190)_ = 825.58, p < 0.001,* η*_p_^2^ = 0.78 that showed that listeners could clearly discriminate among the different vocal sex ratios (see Fig. 1). The linear trend was significant *F*_(1,238)_ = 1016.20, p < 0.001 *η*_p_^2^ = 0.81. There was a significant main effect for talker age indicating that listeners heard a smaller percentage of male voices in the array of young talkers (*M* = 46.0, *SE* = 0.39) than in the array of old talkers (*M* = 48.8, *SE* = 0.39), *F*_(1,476)_ = 37.41, p < 0.001 *η*_p_^2^ = 0.14. Phrased differently, listeners heard a higher proportion of young female voices than old female voices. Listeners significantly overestimated the actual percentage of females for both young, *t*(239) = 10.16, p < 0.001, *d* = 0.66, and old voices *t*(239) = 0.71, p = 0.48, *d* = 0.20.

The main effect for listener sex was not significant *F*_(1,238)_ = 0.06, p = 0.80 *η*_p_^2^ < 0.001. However, consistent with Experiment 1, there was a significant listener sex by ratio interaction *F*_(5,1190)_ = 6.63, p < 0.001 *η*_p_^2^ = 0.03. Post-hoc Bonferroni corrected t-tests showed that for trials with 0% male voices, men had higher perceived sex ratios (*M* = 25.7, *SE* = 1.21) than women did (*M* = 21.1, *SE* = 1.21). However, for trials with 100% male voices, men had lower perceived sex ratio (*M* = 68.4, *SE* = 1.16) than women did (*M* = 73.5, *SE* = 1.16). This shows that women were more accurate at perceiving sex ratios at extremely biased distributions and that men more than women overperceived female voices when they were scarce and underperceived them when they were plentiful.

There was also a significant interaction between sex ratio and talker age, *F*_(5,1190)_ = 28.22, p < 0.001 *η*_p_^2^ = 0.11. The percentage of male voices heard in the sample of old voices was higher than that heard in the young sample at sex ratios above 50%. However, at 0% (when there were no male voices), the perceived number of male voices heard was higher in the older voices. No other interactions were significant.

#### Error rates

A second 2 (talker age) × 2 (listener sex) × 2 (number of talkers) × 6 (sex ratio) mixed design ANOVA was conducted on the absolute error rates for each participant. Absolute error was calculated as in Experiment 1. There was a significant main effect for sex ratio *F*_(5,1190)_ = 281.48, p < 0.001,* η*_p_^2^ = 0.54, that showed higher error rates as actual sex ratios departed from 50% (see Fig. 2). There was a significant main effect for talker age *F*_(1,238)_ = 11.41, p = 0.001 *η*_p_^2^ = 0.05 that showed higher error rates for young voices (*M* = 18.16, *SE* = 0.43) than for old voices (*M* = 17.15, *SE* = 0.41), and a significant main effect for sex *F*_(1,238)_ = 10.46, p = 0.001 *η*_p_^2^ = 0.04 that showed higher error rates for men (*M* = 18.91, *SE* = 0.56) than for women (*M* = 16.38, *SE* = 0.56). As in Experiment 1, each of these main effects was qualified by significant interactions.

**Figure 4:**
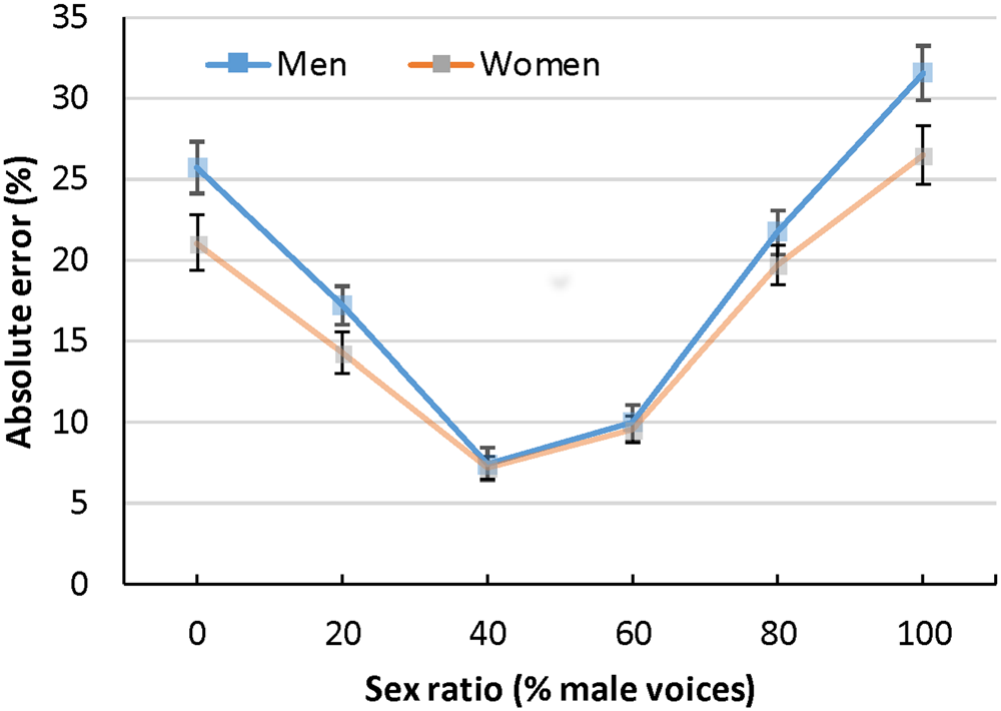
Absolute error rates for men and women in Exp.2 (ten simultaneous voices). Error bars represent 95% confidence intervals.

**Figure 5:**
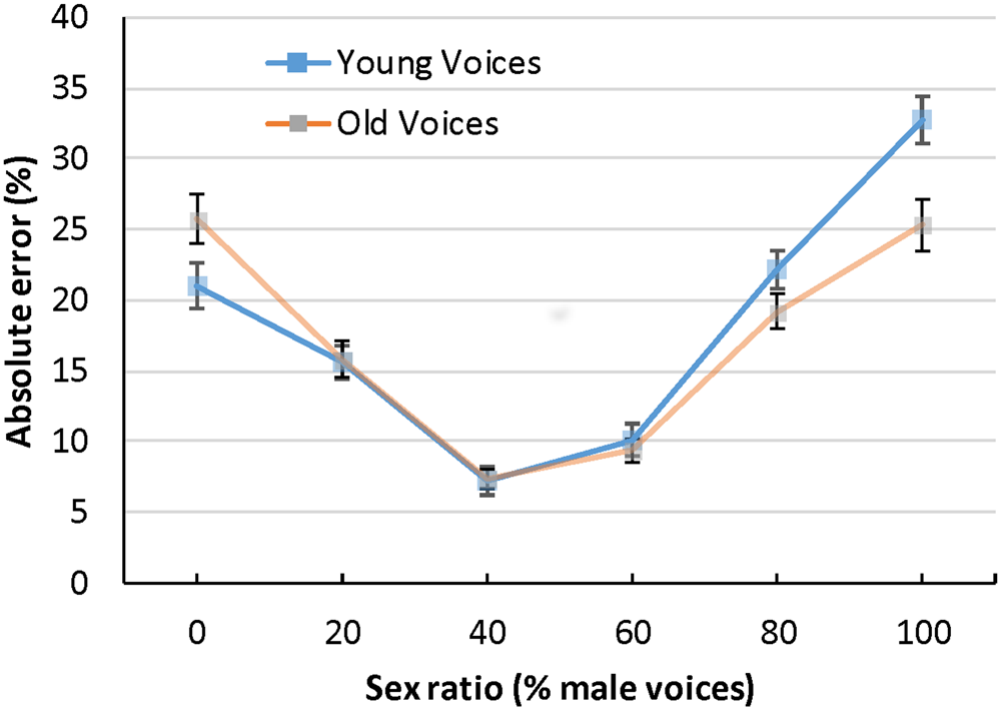
Absolute error rates for young and old voices in Exp.2 (ten simultaneous voices). Error bars represent 95% confidence intervals.

The interaction between sex ratio and listener sex was significant, *F*_(5,1190)_ = 4.33, p = 0.001 *η*_p_^2^ = 0.02. Men and women had similar error rates for sex ratios near 50%. However, women performed better at extreme sex ratios (see Fig. 4). There was also a significant interaction between sex ratio and voice age, *F*_(5,1190)_ = 43.36, p < 0.001 *η*_p_^2^ = 0.15. Listeners had similar error rates for young and old voices near 50% but had higher error rates for young voices when sex ratios were high and lower error rates for young voices when sex ratios were low (see Fig. 5). No other interactions were significant.

### Discussion

The results of Experiment 2 are largely consistent with those of Experiment 1. After brief presentations of ten simultaneous voices, listeners accurately scaled vocal sex ratios. Sex ratios near 50% were perceived more accurately than more extreme ratios; men had higher error rates than women, and there was better accuracy for young voices than for old voices. However, when the percentage of male voices was above 60% or below 40%, scaling sex ratios with ten voices was more difficult, and performance dropped when compared with scaling five voices in Experiment 1. The overall absolute error rate with ten voices was 17.7% was significantly higher than the absolute error rate of 11.9% with five voices in Experiment 1, *t*_(238)_ = 13.02, p < 0.001. Yet, there were no differences in absolute error rates between Experiment 1 and Experiment 2 at 40% or at 60%. Poorer performance at the extremes yielded a lower mean correlation between actual and perceived sex ratios, fewer participants with significant correlations, and smaller effect sizes for all significant findings when listeners heard ten versus five simultaneous voices.

The performance decrement with additional voices might be due to an increase in acoustic masking that would make some cues to vocal sex more difficult to perceive^35,37,101,110^. Although most of the research on multiple talker masking has examined the effects of masking on speech intelligibility of a single talker in the array, increasing the number of simultaneous talkers would likely also mask many cues to vocal sex (e.g., formant spacing, pitch variability, speaking rate, etc.). However, this would not explain why error rates did not differ between Experiments 1 and 2 at 40% and 60%.

The salient effects of young female voices were also replicated in Experiment 2. Because listeners generally underestimated sex ratios, they heard a significantly higher percentage of female voices than actually were present. There was also a significant interaction between ratio and voice age; more female voices were heard at extreme ratios in the young sample than in the old sample. At intermediate sex ratios closer to 50%, there was no difference between old and young voices (though the percentage of female voices was still overestimated). Listener sex interacted with ratio in a similar manner at the extremes. When sex ratios were high, women heard more males voices than men did. When sex ratios were low, this pattern was reversed. This resulted in better overall performance by women in scaling sex ratios, supporting previous work that shows a female advantage in voice processing and ensemble coding^18,50,52,53,55,92^.

## General Discussion

Sex ratios are correlated with a wide variety of sociosexual behaviors in a diverse array of species^8^. Thus, it is reasonable to assume that observers have some means of detecting sex ratio information in a local population. The current work shows that listeners can extract operational sex ratios from briefly presented vocal stimuli and that perceived sex ratios depend upon the sex of the listener, and the age of the voices heard. The results provide the first evidence for human vocal coding of sex ratios and may have corresponding sociosexual implications for behavior in any local environment in which sex ratios are unbalanced.

Because of the crucial relationships between sex ratios and sexual selection, it is likely that detecting sex ratio information is an automatic rather than an effortful process. For example, Watkins^5^ showed that brief exposures to slideshows of faces depicting unbalanced sex ratios immediately influenced preferences for facial symmetry. Listeners in the current study scaled sex ratio information accurately after exposures of only 1500 ms which is largely consistent with work showing that observers can visually scale sex ratios of faces after brief exposures of 330 ms^15^. Thus, although observers may build stable cognitive representations of local sex ratios over time, the current evidence suggests that sex ratio information is also immediately available.

Increasing the number of voices in the array from five to ten increased the overall absolute error in scaling sex ratios. However, this increase in error only occurred when the percentage of males was above 60% and below 40%. In the range of sex ratios that are more typical of those that occur naturally, there was no difference in error rates between five and ten voices. Although the specific acoustic cues that listeners use to determine vocal sex ratios are currently unknown, acoustic masking of some of the cues to vocal sex could be increased with an increasing number of voices. This might force a greater reliance on fundamental frequency, and listeners have been shown to be able to use ensemble processing to determine mean frequency of a group of tones^88^. However, the failure to find any differences in error rates at 40% and 60% suggests that listeners may also be tuned (perhaps both phylogenetically and ontogenetically) to make more accurate estimations of sex ratios when presented with those that occur more frequently. Previous exposure to relatively balanced sex ratios may also set up perceptual expectations that sex ratios are close to 50%. However, the significant positive correlations between actual and perceived sex ratios for the overwhelming majority of participants suggests that it is not likely that listeners simply responded near 50% regardless of the actual sex ratio presented.

### Sex Differences in Perceived Sex Ratios

Across both experiments, women showed better performance than men in processing sex ratios, particularly because of better performance at the extremes. Access to fundamental frequency information may partially explain this difference. Watkins^55^ used a spectral filtering technique to show that the low versus high-frequency components of male voices play a crucial role for voice processing by women, but that men do not differ in their reliance on high versus low-frequency information in processing male voices. Thus, women might be able to leverage this sensitivity when judging vocal sex ratios and show greater accuracy than men based better detection and processing of male voices. The advantage that women showed in perceiving vocal sex ratios is also consistent with work that shows that women are more sensitive than men to the social cues present in voices^54^. In particular, women show better sensitivity to dominance cues in voices than men do^18,50^. Importantly, “dominance” in this context was manipulated as differences in fundamental frequency with lower frequency voices perceived as more dominant.

### Voice Age and Operational Sex Ratios

In both experiments, listeners heard more male voices in the old voices than in the young voices. This suggests that listeners are differentiating operational sex ratios from adult sex ratios. The fundamental frequency of the female voice drops with age and is correlated with a drop in female fertility over the lifespan^66,82^. Although there may be psychoacoustic reasons why more male voices are heard in the old sample of voices (perhaps a higher similarity of male and female voices in the elderly), it is important to note that listeners are sensitive to these cues and that they influence perceived sex vocal ratios differentially for men and women. This is finding is consistent with previous work that has shown processing advantages for young female voices over male voices^111,112^.

### Conclusions and Limitations

That listeners can accurately scale sex ratios for up to ten briefly presented simultaneous voices with exposures of only 1500 ms suggests that auditory processing of sex ratios is an automatic process. Although these types of brief stimuli would rarely occur in a natural environment, they likely tap into sex ratio processing mechanisms that are nonetheless, active in more naturalistic situations. Because the data were collected online, there was wide variability in the specific devices that presented the stimuli and collected the responses. However, it is important to note that this increased variability works against the current research hypothesis making it less likely to reject the null hypothesis. Finding and replicating significant results in light of this increased variability speaks to the robust nature of the effects. Thus, in a natural environment, listeners may be even more accurate at scaling sex ratios than is indicated by the current results. This point is further reinforced by the fact that there were no spatial cues present in the stimuli. Spatial separation of voices can improve the perception of individual voices and thus, may also lead to improved perception of sex ratios by voice^113,114^.

Future investigations of vocal sex ratios might examine the specific acoustic characteristics that are important for accurate auditory perception of sex ratios. For example, the spectral filtering approach to voice perception used by Watkins^55^ could be employed to examine how manipulating high versus low-frequency cues influence perceived sex ratios. Listeners might also be presented with a series of single voices over time instead of simultaneous voices. This method would provide information on how cognitive representations of sex ratios are encoded and retained over time. A two-alternative-forced-choice method might also be employed in which listeners are presented with two different sex ratios in succession and make a forced choice as to which sample contains more male voices. This would provide greater insight into the precision with which listeners can estimate auditory sex ratios.

In a broader sense, the results support a theoretical position that suggests perception of the local environment is not veridical^115^. Rather, it has been shaped by evolution to provide a representation that better enables successful survival and reproduction^97,115,116^. Individual differences that impact successful survival and reproduction shape subjective experience of the external world.
